# October Notices

**Published:** 1864-10

**Authors:** 


### OCTOBER NOTICES.

In these days of war, people say that every thing rises except the cream on milk served us every day from elegantly painted milk carts "illustrated", with cows having large, long and bushy tails, as a proof that said milkman never feeds swill on his premises. Now as cow tails will rot off when fed on swill, and are kept until they fall down dead, after the last milking of an hour before, in their miserable, dark, filthy and noisome stalls, it "stands to reason," that a cart which has painted on it a fine, healthy farm house cow, with as grand a tail as any body could wish to see, cant possibly sell swill milk ; because the cow on the cart pannel has a tail, whoever "don't see" that, has got no sight at all and ought to get a set of glass eyes or a pair of leathern spectacles. But there are exceptions to all general rules ; cream does not only rise now but always has risen on the milk served by the Rockland County and New Jersey Milk Association at 146 East 10th Street and corner of Broadway and 37th Street, under the energetic and watchful superintendence of Mr. S. W. Canfield, General Agent for the Company.

Patriotism.—Every intelligent patriot and respectable citizen ought to patronize "The Citizen," published weekly at 813 Broadway, by an association of gentlemen, at three dollars a year. Its first great object is to reduce the taxes, which ought and can be done by the simple plan of electing men to fill all the city offices who are known and acknowledged by all parties to be men of tried honesty, whether they be rich or poor or of whatever political party ; the first questions to be proposed to every candidate are,

IS HE HONEST ?

IS HE CAPABLE ?

The poor of New York are the class most deeply interested in having good men and true to administer the city government, for let such bear in mind always that they are the men who really pay the taxes, for if a house owner is taxed three dollars instead of one, on a hundred, as to the valuation of his house, he adds the two dollars to his rent, and those who rent have to pay it. And it is the large hearted men of the city who sympathize truly and practically with the poor in the great burdens now imposed upon them, who are endeavoring to diffuse inormation, through "The Citizen," which is calculated to enlighten all classes on this most important and practical subject.

A report of the sanitary condition of the city during 1863 has been sent to us by F. J. A. Boole, Esq., the most energetic, thorough and efficient city inspector we have had in many years, and as such, he merits the confidence, respect and support of every good citizen.

Central Park.—We are indebted to the courtesy of George M. Van Nort, Esq., for the 7th Annual report of the Board of Commissioners, up to January 1st, 1864. The Central Park of 844 acres, has cost the city seven and three-quarter millions of dollars. There are completed six miles of road for horseback riding ; seven of carriage driving, and twenty of walks. The Park was begun in 1859. The skating pond, for rowing in summer, is twenty acres in extent, four feet deep. The smaller pond near 59th street is four acres. New reservoir 106 acres, thirty-six feet deep, costing one-and-a-half millions of dollars, and holds a thousand million gallons of water, constructed in three years. Skating begins about Christmas and ends in February. There were 19 skating days the first winter, 38 the second, 27 the third, 50 the fourth and eleven last year up to December 31, 1863. The art of skating was first practised in St. James Park, London, two hundred years ago, to 27th December 1862 ; the "runners" then were bones attached to the feet. The music days in the Park are from nine to twenty days. Rowing on the 20 acre pond is the great amusement for summer at thirty cents for half an hour for one or two persons, and ten cents additional for each one in addition ; the circuit of the lake, a distance of two miles is accomplished in half an hour ; for moonlight of a summer evening it is lovely ; and then the water fountain and the cassino for ice cream, &c., on your way home, at moderate charge, or coffee, or a good supper, hot on the spot. During 1863 177,000 vehicles entered the Park, 16,000 equestrians and over half a million on foot. The average Sunday attendance at the park is fifteen thousand, most in August, next in May. The greatest number visit the Park from 3 to 4 P. M., a third of a million in a year ; from 4 to 5 next ; less than 2000, (of all the four-and-a-half millions) before 6 A. M., and about as many from 10 to 11 P. M. The largest number of Pedestrians who visited the Park during any one day was on Christmas of 1863, being over 94,000, and only a hundred and six December 17, 1863. The largest number of vehicles for one day was June 27th, near 10,000 A quarter of a million of trees and shrubs have been planted. The Park was laid out by a native of Hartford, Connecticut. The city does not pay a dollar of the interest on the money expended on the Park because the land adjoining the Park has increased enough in value to make the assessment more than pay the interest ; that is, the men pay the interest on the Park debt whose lands adjoin it and which on that account have increased so much in value. Fifth and Eighth Avenues front the Park ; not a house has been built on Fifth Avenue opposite the Park south of 70th Street, nor can a lot of twenty feet front, running back 100 feet be purchased for less than twelve or fifteen thousand dollars ; that section is designed to be the "west end" of the great metropolis of the nation. Every person who wishes to entertain friends who may visit the Park together, should impress the above statements indellibly on the memory ; they are just the items which every intelligent stranger feels an interest in knowing.

Taxation.—One among other benefits of increased taxes is that in New York City a million of cigars were made daily by two thousand workmen with wages averaging two dollars a day; since the tax, the manufacture returns show a decrease of one half. Another benefit is that high taxes compel economy, habitual economy, a virtue which influences the whole character. The world over, the economical are industrious, thrifty and trustworthy, and what many may not have observed, economical persons are very apt to be tidy, tasteful and cleanly ; it saves work to repair clothing, tools or anything else ; it saves work to keep the clothing clean, as a noble hearted little fellow once said to a bystander who had witnessed another boy throwing mud on his clean and well patched trowsers, "why don't you throw mud on him." "Then there would be two suits of clothes to wash." Notice that answer, you thoughtless dowdy and "mussy" daughters, girls and young ladies who take no pains to preserve your clothing from being soiled, on the ground that the "servants are paid to work." Among the first duties of a young wife is to learn how to save in housekeeping, while her generous husband is trying to lay up at his workshop, his store, or his banking house. And when a youth begins to "save," to lay up money, he is "saved" from a life of idleness, and crime.

"The Church and the Rebellion ;" 'A consideration of the Rebellion against the Government of the United States, and the agency of the Church, North and South, in relation thereto.' By R. L. Stanton, D. D., Professor in the Presbyterian Theological Seminary at Danville, Ky. Dedicated to the young men of the United States of every creed in religion and every party in politics. Published by Derby and Miller, New York, 592 pp., $2.00.—Dr. Stanton is an able scholar, a logical writer and a fearless controversialist. He utters his sentiments in language at once clear, concise and forcible. You always know the side he takes. The book contains valuable historical and documentary evidence which will make it a standard and reliable work of reference. The main points are four :

1st. The Rebellion is the Work of Traitors.

2nd. Slavery is the cause of the Rebellion.

3rd. The leading Southern clergy are responsible for the war.

4th. That a glorious future awaits us in an undivided country in which "The sun shall no more rise upon a master nor set upon a slave."

The language of the book is often sublime. The last subjects are God reigns, The Patriot's Reward and the Traitors Doom. The last sentence—"THEN LET THE MEMORY OF THE WICKED ROT" ! !

GYMNASTICS.—Clerks, sedentary persons and families, can obtain the highest advantages of gymnastic training at Mrs. Plumb's well ordered establishment in 14th Street, New-York.

UNIVERSITY OF MICHIGAN.—Its catalogue for 1864 shows the institution at Ann Arbor to be in a very prosperous condition. The only charges for instruction are Ten Dollars admission fee and five dollars per annum thereafter. Board in private families from two to three dollars a week. A notice able feature is the establishment of a chair of Physiology and Hygiene to which A. B. Palmer, A. M., M. D. formerly Prof. of same in the Berkshire Medical College at Pittsfield, Mass., has been elected and has entered on the duties of his office. It seems then at long last, it is beginning to be felt of some importance to teach the young how to take care of their health, before it is irrecoverably ruined.. Prof. P. besides his acknowledged ability, brings a characteristic enthusiasm, energy and thoroughness into the discharge of his very important duties in the introduction of sanitary science as a distinct branch, into colleges and seminaries of learning.

